# Deep Learning Meets InSAR for Infrastructure Monitoring: A Systematic Review of Models, Applications, and Challenges

**DOI:** 10.3390/s25237169

**Published:** 2025-11-24

**Authors:** Miguel Fontes, Matúš Bakoň, António Cunha, Joaquim J. Sousa

**Affiliations:** 1 Engineering Department, School of Science and Technology, University of Trás-os-Montes e Alto Douro, 5000-801 Vila Real, Portugal; acunha@utad.pt (A.C.); jjsousa@utad.pt (J.J.S.); 2 Centre for Robotics in Industry and Intelligent Systems (CRIIS), Institute for Systems and Computer Engineering, Technology and Science (INESC-TEC), 4200-465 Porto, Portugal; 3 Insar.sk Ltd., Konstantinova 3, 08001 Presov, Slovakia; matusbakon@insar.sk; 4 Department of Finance, Accounting and Mathematical Methods, Faculty of Management and Business, University of Presov, Konstantinova 16, 08001 Presov, Slovakia; 5 ALGORITMI Research Centre, University of Minho, 4800-058 Guimaraes, Portugal

**Keywords:** Deep Learning, InSAR, infrastructure monitoring, systematic review

## Abstract

Monitoring civil infrastructure is increasingly critical due to aging assets, urban expansion, and the need for early detection of structural instabilities. Interferometric Synthetic Aperture Radar (InSAR) offers high-resolution, all-weather surface deformation monitoring capabilities, which are being enhanced by recent advances in Deep Learning (DL). Despite growing interest, the existing literature lacks a comprehensive synthesis of how DL models are applied specifically to infrastructure monitoring using InSAR data. This review addresses this gap by systematically analyzing 67 peer-reviewed articles published between 2020 and February 2025. We examine the DL architectures employed, ranging from LSTMs and CNNs to Transformer-based and hybrid models, and assess their integration within various stages of the InSAR monitoring pipeline, including pre-processing, temporal analysis, segmentation, prediction, and risk classification. Our findings reveal a predominance of LSTM and CNN-based approaches, limited exploration of pre-processing tasks, and a focus on urban and linear infrastructures. We identify methodological challenges such as data sparsity, low coherence, and lack of standard benchmarks, and we highlight emerging trends including hybrid architectures, attention mechanisms, end-to-end pipelines, and data fusion with exogenous sources. The review concludes by outlining key research opportunities, such as enhancing model explainability, expanding applications to underexplored infrastructure types, and integrating DL-InSAR workflows into operational structural health monitoring systems.

## 1. Introduction

The monitoring of civil infrastructure, including dams, bridges, highways, and urban systems, has become increasingly important due to concerns over structural integrity, public safety, and potential economic consequences of failures. Undetected degradation can lead to critical incidents with severe human and financial impacts. As infrastructure systems age and urban areas expand globally, the demand for scalable and timely monitoring solutions has grown substantially.

Conventional monitoring techniques, such as in situ inspections, topographic surveys, and ground-based sensors (e.g., accelerometers and strain gauges), remain valuable but are limited by high operational costs, restricted spatial coverage, and low temporal resolution [1,2,3,4]. Although recent developments in ground-based sensing and AI-assisted unmanned aerial vehicle (UAV) visual inspections offer improved automation and flexibility [5], these approaches often lack the scalability and continuity required for monitoring large or inaccessible infrastructure networks [6].

Remote sensing technologies have emerged as a viable alternative for continuous infrastructure monitoring at larger scales [7]. While optical satellite imagery supports the identification of surface changes, it is constrained by weather conditions and illumination [8]. In contrast, Synthetic Aperture Radar (SAR) enables data acquisition under all-weather, day-and-night conditions, supporting the precise detection of surface deformation [9,10]. Among SAR-based methods, Interferometric Synthetic Aperture Radar (InSAR) is particularly effective for capturing sub-centimetric to millimetric displacements over time, making it suitable for monitoring structural stability [11,12]. InSAR offers extensive spatial coverage and does not require on-site data collection, which is advantageous in hazardous or remote areas. Nevertheless, interpretation of InSAR data remains challenging due to atmospheric artifacts, noise from decorrelation, and difficulties in distinguishing actual displacements from measurement errors [13,14].

The integration of Deep Learning (DL) techniques has improved the analysis and interpretation of remote sensing data, including InSAR. Convolutional Neural Networks (CNNs), Recurrent Neural Networks (e.g., LSTMs), and Transformer-based models have been applied to various stages of the infrastructure monitoring workflow, from pre-processing to deformation prediction and risk assessment [15,16,17,18]. These approaches have been investigated in contexts such as urban areas, dams, transport infrastructure, and power grids. DL models offer the potential to automate processes and handle large-scale, multi-temporal data. However, limitations remain, including the scarcity of labeled datasets, difficulties in model generalization across environments, and the lack of interpretability in many DL applications [19].

Despite increased interest in combining DL and remote sensing, existing literature reviews often focus either on broader applications of DL in SAR or environmental monitoring, with limited attention to the specific challenges associated with infrastructure monitoring using InSAR [20,21]. This gap in the literature is significant, given that the combination of DL and InSAR presents domain-specific challenges, such as data heterogeneity, complex temporal patterns, and operational constraints, that merit focused investigation. A structured review is therefore essential to consolidate current knowledge, evaluate methodological approaches, and identify directions for future research.

Given the increasing reliance on data-driven tools in infrastructure asset management and the strategic relevance of preventive monitoring, this review provides timely guidance for bridging scientific research and operational practice.

This article addresses that need by presenting a systematic and critical review of DL techniques applied to infrastructure monitoring using InSAR data. The review synthesizes existing methods, discusses their strengths and limitations, and highlights the principal technical and operational challenges that remain unresolved. In addition, the article outlines avenues for future research, with emphasis on robustness, explainability, and applicability to real-world scenarios. The review is based on a structured selection protocol, described in Section 3, which ensures reproducibility and methodological transparency.

The main contributions of this review are:

A systematic synthesis of DL techniques applied to InSAR-based infrastructure monitoring;

An analysis of the key methodological challenges and practical limitations;

A comparative overview of current approaches, highlighting research trends, gaps, and future opportunities.

## 2. Materials and Methods

### 2.1. Existing Systematic Reviews

Despite growing interest in combining DL and InSAR for Earth observation applications, the number of systematic reviews specifically addressing infrastructure monitoring remains limited. Existing surveys tend to focus on individual technical aspects or natural phenomena, with little integration or synthesis relevant to structural engineering contexts.

Some reviews address DL applications in geological risk analysis, where InSAR plays a prominent role in detecting surface deformation related to earthquakes, volcanic activity, and landslides [22]. While convolutional networks have been used in these contexts, the focus remains on natural environments, without direct extrapolation to artificial structures.

Other contributions explore DL for InSAR time series processing, highlighting the use of architectures such as U-Net with attention mechanisms and generative adversarial networks for atmospheric noise reduction [23]. Although technically innovative, these studies do not extend their scope to structural or infrastructure monitoring scenarios.

Methodological reviews also exist that focus on challenges such as phase unwrapping, where DL is investigated as a tool to improve the quality of interferometric products [24]. However, these remain at the signal processing level, with no direct connection to practical applications in structural surveillance.

Some reviews examine the integration of InSAR and DL within broader multisensor change detection frameworks [25]. These contributions are generally concerned with environmental and land use dynamics, rather than with technical inspection or maintenance of physical infrastructure.

Slope monitoring and landslide forecasting are common topics, with InSAR used to assess geotechnical instability [26]. Nonetheless, these methods are typically anchored in natural terrain analysis and rarely translated to infrastructure-specific settings.

More general reviews on the use of convolutional networks with SAR data occasionally reference InSAR for tasks such as speckle filtering or terrain classification [27]. However, these do not analyze the potential of combining DL and InSAR in targeted structural monitoring contexts.

The set of reviews analyzed confirms significant advances in technical subdomains but reveals a fragmented and poorly articulated approach to integrating DL and InSAR in infrastructure engineering. The absence of a critical and practical application-oriented perspective compromises the usefulness of existing knowledge. In this sense, developing a review that, in addition to organizing and clarifying the state of the art, establishes research priorities and contributes to consolidating solutions with real applicability in the field is justified.

### 2.2. Identified Gaps

The intersection between DL and InSAR in the specific context of infrastructure monitoring remains underexplored. Most reviews prioritize topics such as natural hazard assessment or core interferometric processing challenges, e.g., atmospheric correction, phase unwrapping, or noise mitigation, without addressing the use of DL models to monitor structural assets such as bridges, dams, or tunnels.

There is limited analysis of how DL has been applied to detect or quantify structural deformation. Few works explore which neural network architectures are most common, the types of deformations considered, or the criteria used to evaluate performance. Comparative studies between different classes of models, such as convolutional, recurrent, or attention-based, are rare, limiting a broader understanding of their suitability for infrastructure-related applications.

Technical challenges inherent to InSAR data, such as speckle noise, atmospheric delay, and phase ambiguity, are often treated in isolation. Reviews rarely discuss how these factors affect DL model performance or how they are handled in training and inference processes.

Model interpretability is another neglected area. In structural engineering contexts, the ability to understand and justify model outputs is essential, particularly when these influence safety-critical decisions. However, few reviews address techniques for explainability or the trustworthiness of DL predictions in infrastructure monitoring scenarios.

Furthermore, there is a lack of documented case studies that demonstrate practical, real-world applications of DL-InSAR integration in operational infrastructure settings. Situations such as subsidence in urban areas, slope failures near transportation corridors, or post-seismic impact assessments are seldom included. This restricts the assessment of the maturity and readiness of current approaches. Open challenges such as the scarcity of labeled datasets, the need for generalizable models, and the integration of physical priors are also underrepresented.

These observations confirm the need for a focused and structured review that consolidates existing evidence and provides a domain-specific analysis of how DL and InSAR are jointly applied in infrastructure monitoring.

### 2.3. Contribution of This Review

The current literature presents relevant advances in the use of DL with InSAR data, but does not include a review centered on its application to monitoring built infrastructures. This gap justifies a systematic analysis focused on this area, to map existing approaches, the architectures used and the specific challenges of this application.

The proposed review aims to combine studies that apply DL to InSAR data for detecting and monitoring structural deformations, identifying the methods used, the application contexts and the most frequent limitations. It also aims to highlight the lack of studies applied to real scenarios and the limited attention paid to the interpretability of the models. Our contribution is threefold:

(i) we systematize the literature on DL-InSAR applications in infrastructure monitoring, identifying trends and methodological patterns;

(ii) we examine the architectures most commonly used, their roles within different stages of the monitoring pipeline, and their strengths and limitations.

(iii) we highlight the limited number of real-world applications and the scarcity of research on model interpretability, pointing to concrete directions for future research.

By addressing these elements, this review offers a consolidated foundation for developing more effective, scalable, and trustworthy monitoring systems that combine DL with InSAR.

## 3. Methodology

This review adopts a systematic approach to identify, select, and analyze scientific studies focused on the application of DL models to infrastructure monitoring using InSAR data. The methodology follows established guidelines for conducting reproducible and comprehensive literature reviews and is structured into four main components: research questions, data sources, inclusion/exclusion criteria, and article selection strategy. This review was not prospectively registered and no formal review protocol was prepared.

### 3.1. Research Questions

This systematic review is structured around three central research questions (RQs), formulated to address identified lacunae in the current literature and the specific challenges inherent in applying DL methodologies to Interferometric Synthetic Aperture Radar (InSAR) data for infrastructure monitoring.

Firstly, despite the proliferation of DL applications within the broader field of remote sensing, a systematic synthesis detailing the specific architectures adapted or developed for InSAR-based infrastructure monitoring is currently lacking. To address this, the review investigates (RQ1) the landscape of DL models employed in this domain. This involves identifying prevalent architectures, critically evaluating their reported strengths and limitations within this specific application context, and discerning potential trends in model selection relative to monitoring tasks or infrastructure typologies. Furthermore, this question assesses the alignment between deployed models and the intrinsic challenges posed by InSAR data, such as spatial sparsity and temporal irregularity.

Secondly, the integration points of DL within the InSAR processing and analysis workflow—ranging from atmospheric noise mitigation to deformation forecasting—remain poorly characterized. Understanding the distribution of DL applications across the monitoring pipeline is essential for identifying methodological gaps and opportunities for innovation. Therefore, this study examines (RQ2) the specific stages within the InSAR monitoring pipeline where DL techniques are predominantly integrated. This analysis aims to map common entry points, reveal under-explored phases with potential for advancement, and evaluate the extent to which DL contributes to the automation and refinement of established InSAR analysis protocols.

Thirdly, while the synergy between DL and InSAR offers substantial potential for monitoring complex or inaccessible infrastructure, the thematic distribution of its application across different infrastructure classes is not well documented. Characterizing this distribution is crucial for identifying scenarios where these technologies provide maximal value and highlighting critical infrastructure types where adoption, although potentially beneficial, remains limited. Consequently, this review addresses (RQ3) the types of infrastructure most frequently subjected to DL-InSAR monitoring. This analysis seeks to provide empirical grounding for future research prioritization and the development of targeted, operationally relevant applications.

### 3.2. Data Sources

We selected the following databases for our review: Scopus, IEEE Xplore, and others that provide strong coverage in engineering, remote sensing, and computer science. These databases were chosen due to their comprehensive coverage in the relevant interdisciplinary fields, ensuring access to a broad spectrum of studies while minimizing redundancy.

While we considered including databases such as Web of Science and ScienceDirect, preliminary searches revealed a significant overlap in the studies indexed by the chosen sources. Most of the articles relevant to this review were already captured by Scopus and IEEE Xplore, which are well-established in indexing literature from the engineering and remote sensing fields. We thus determined that the selected databases were sufficiently comprehensive for the scope of this review, allowing us to streamline the search process and avoid unnecessary duplication in indexing. This selection ensures that the review captures all pertinent studies without bias toward any particular database or access model.

### 3.3. Inclusion and Exclusion Criteria

The inclusion and exclusion criteria definition establishes the parameters for selecting studies to be considered in the review. These criteria were used to ensure that only articles related to applying DL to InSAR data in infrastructure monitoring were included in the analysis.

**Inclusion Criteria:**

- Articles that apply methodologies based on deep neural networks.
- Articles in which InSAR data is used directly as input for DL models, without being limited to validation, comparison or visualisation functions.
- Articles that use InSAR data or derived techniques, such as PSInSAR, SBAS or DInSAR.
- Articles addressing the monitoring of infrastructure at risk or phenomena with a potential impact on infrastructure.

**Exclusion Criteria:**

- Articles that only use traditional Machine Learning techniques, without resorting to deep neural networks.
- Articles using InSAR data only for validation or visual support, but not as direct input into DL models.
- Articles that do not use InSAR data or associated techniques.
- Articles that do not monitor infrastructure or establish a relationship with infrastructure risk.
- Duplicate articles that do not contribute directly to the review topic.

### 3.4. Search and Selection Strategy

The article selection followed a four-step protocol designed to ensure methodological transparency and reproducibility:

**Step 1—Initial Search:** An initial search was conducted across the selected databases (Section 3.2). To capture relevant literature, a comprehensive Boolean query was designed to identify studies at the intersection of three core thematic domains: Deep Learning, InSAR, and infrastructure monitoring. The query combined extensive lists of keywords and synonyms for each domain using the ‘AND’ operator, applied to the title, abstract, and keyword fields (TITLE-ABS-KEY).

- The Deep Learning domain included terms such as “deep learning”, “machine learning”, “artificial intelligence”, specific architectures like “convolutional neural network” (and “CNN”), “recurrent neural network” (and “RNN”), “LSTM”, “GRU”, and “transformer”.
- The InSAR domain covered general terms like “InSAR” and “interferometric synthetic aperture radar”, as well as specific techniques such as “PSInSAR”, “DInSAR”, and “SBAS”.
- The Infrastructure Monitoring domain encompassed keywords related to infrastructure types (“infrastructure”, “building”, “bridge”, “dam”, “railway”, “road”), monitoring practices (“structural health monitoring”, “SHM”, “infrastructure monitoring”), and relevant phenomena (“geotechnical”, “landslide”, “slope stability”).

The search was restricted to English-language publications dated between 2020 and February 2025 to ensure the recency and relevance of the reviewed literature.

**Step 2—Duplicate Removal:** Articles retrieved from multiple databases were screened, and duplicates were removed using reference management software.

**Steps 3 and 4—Title/Abstract Screening and Full-Text Review:** Screening and eligibility assessments were conducted by a single reviewer following predefined inclusion and exclusion criteria. The subsequent steps involved qualitative filtering. First, titles and abstracts were screened to exclude articles not directly related to the application of DL techniques to InSAR data for infrastructure monitoring. Following this, a full-text review was conducted on the shortlisted articles to eliminate studies that, although tangentially related, did not fully meet the defined inclusion criteria (Section 3.3). This ensured that only studies making direct contributions to the review’s objectives were included in the final analysis.

This structured process resulted in a final set of studies focused specifically on the integration of DL and InSAR for infrastructure monitoring, providing a solid basis for the subsequent analysis.

### 3.5. Extraction of Study Characteristics

Each article was analyzed to identify the type of InSAR technique used, the DL architecture applied with the main results obtained, the stage of the monitoring process, the infrastructure monitored and the associated phenomenon. Data extraction was conducted by a single reviewer following a predefined extraction template.

The type of InSAR technique was characterized by the use of variants such as conventional InSAR, PSInSAR, SBAS-InSAR, or DInSAR.

The DL architecture described in each article included models such as CNN, LSTM or combinations of different networks, and the main results reported were also summarized. The monitoring stage refers to the phase of the InSAR pipeline in which the model was applied, such as pre-processing, detection, prediction, or evaluation. The monitored infrastructure and the associated phenomenon were also recorded, considering structures such as dams or bridges and events such as landslides or subsidence.

The information extracted from each article was organized into a table to support comparative analysis and the identification of trends in the literature.

## 4. Results

**Figure 1:**
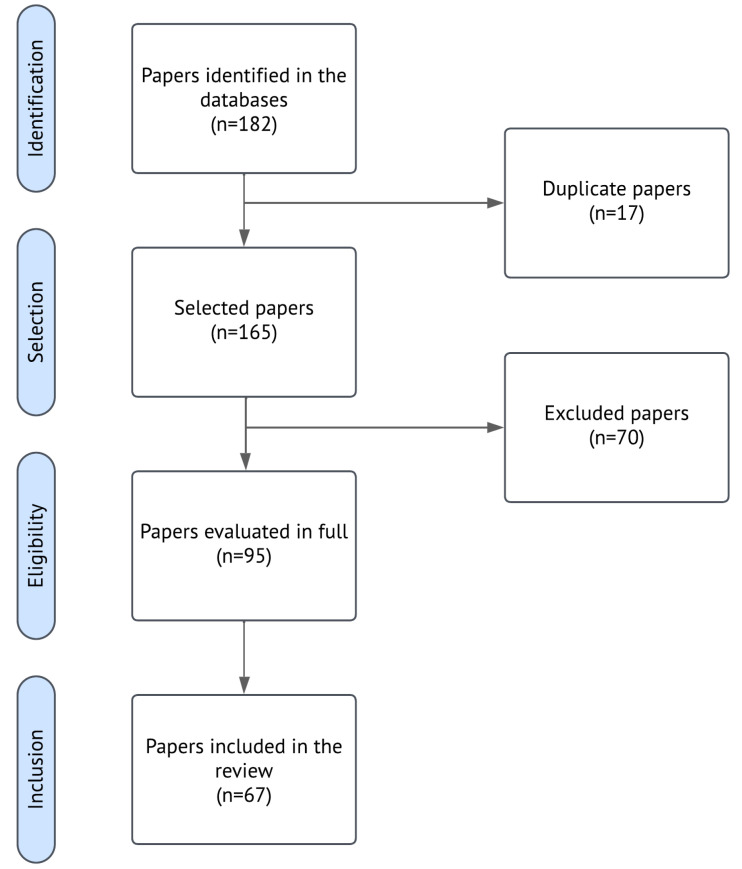
Flow diagram of the identification, screening, and selection of studies. This diagram outlines the systematic review process, following the PRISMA methodology: initial identification through database searches, duplicate removal, title/abstract screening, and full-text eligibility assessment.

This section presents the results of the systematic literature review on the application of DL techniques to infrastructure monitoring using InSAR data. After the inclusion and exclusion criteria (Section 3) were applied, a total of 67 articles were selected for full analysis. Given methodological heterogeneity and the lack of comparable effect measures across studies, a qualitative thematic (narrative) synthesis was conducted, with findings organized by DL architecture, InSAR pipeline stage, and infrastructure type; no quantitative pooling or heterogeneity modeling was performed. The results are structured around the three research questions, emphasizing (i) the DL architectures adopted, (ii) the stages of the InSAR monitoring pipeline to which these models were applied, and (iii) the types of infrastructure most commonly addressed. Supporting data are provided in Figure 1 (PRISMA flow diagram).

After completing the screening and eligibility steps illustrated in Figure 1, a total of 67 studies were selected for in-depth analysis. To support the subsequent discussion, Table 1 summarizes the key characteristics of these studies, including the DL architecture used, type of InSAR technique, infrastructure monitored, and target phenomenon. This synthesis provides a comprehensive view of the methodological diversity and application contexts observed in the literature and serves as the empirical foundation for the analysis developed in Section 4.1, Section 4.2 and Section 4.3.

### 4.1. DL Models Used in Infrastructure Monitoring with InSAR (RQ1)

**Figure 2:**
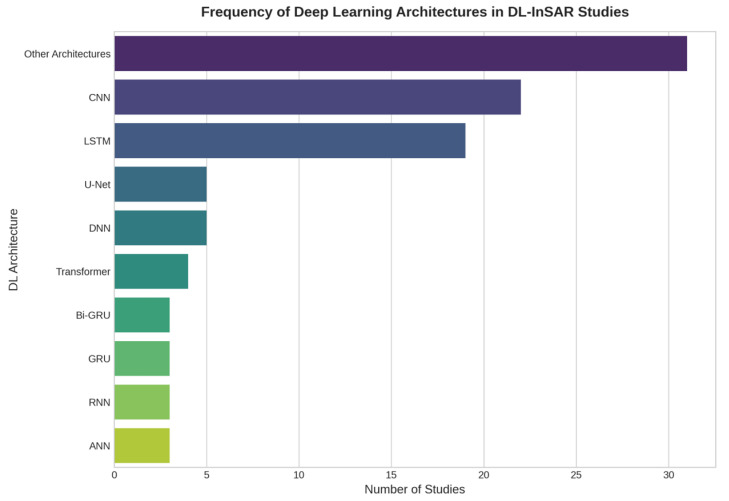
Frequency of DL architectures aggregated from the 67 analyzed studies. The chart illustrates the predominance of CNN and LSTM models, with architectures having two or fewer mentions grouped into the ‘Other Architectures’ category.

To quantify the methodological trends in DL model selection, Figure 2 illustrates the frequency of different neural network architectures adopted across the reviewed studies. The analysis shows a clear predominance of LSTM networks for time-series modeling, CNNs for spatial analysis, and U-Net variants for segmentation tasks.

#### 4.1.1. Frequency and Diversity of Architectures

The analysis reveals a clear concentration on a few core architectures, as quantified in Figure 2. CNNs and Recurrent Neural Networks (RNNs), particularly Long Short-Term Memory (LSTM), are the dominant models. LSTMs appear in 19 studies (approximately 28% of the corpus) and are the standard choice for time-series modeling and deformation prediction [28,29,51,58,77,93]. CNNs, found in 23 studies (34%), are primarily used for spatial tasks, either as standalone models for feature extraction [36,44,52] or as part of hybrid configurations [31,39,40]. A specific and frequent application of CNNs is for segmentation, where U-Net-based architectures (5 studies, 7%), often enhanced with attention mechanisms, are applied in spatial detection tasks [42,45,63,85].

#### 4.1.2. Hybrid and Emerging Architectures

Beyond these core models, a growing number of studies implement hybrid architectures that combine CNNs with LSTMs or GRUs [31,39,47], representing about 10% (7 studies, see Figure 2) of the reviewed literature. For instance, studies [40,62] employ CNN + Bi-GRU configurations to improve temporal pattern recognition. Transformer-based models (5 studies, 7%) are also gaining ground for long-sequence modeling [84,90]. In the most complex implementations, multi-component pipelines integrate segmentation, detection, and prediction modules (e.g., U-Net + YOLOv3 + DnCNN) in a cohesive workflow [30,85].

#### 4.1.3. Less Common but Noteworthy Models

Finally, other architectures appear less frequently. Simpler feedforward networks like DNNs, MLPs, and ANNs (totaling 8 studies, 12%) are used in susceptibility classification tasks [44,57,71]. For highly precise spatial segmentation, Mask R-CNN has been applied (2 studies) [34,86]. Emerging non-convolutional approaches include graph-based networks [56] and Self-Attention Models (SAM) [94], found in single instances within this review.

### 4.2. DL Applications in InSAR Data Processing (RQ2)

To ensure a comprehensive understanding of how DL enhances InSAR-based infrastructure monitoring, it is essential to distinguish between two primary application areas: InSAR data processing and the subsequent monitoring/analysis tasks. While data processing involves preparing and enhancing raw InSAR data, such as atmospheric noise correction, phase unwrapping, and interpolation, monitoring and analysis tasks are concerned with extracting actionable insights, such as detecting temporal deformation patterns and segmenting affected infrastructure. These stages are closely interrelated, as the accuracy and quality of the processed data directly influence the effectiveness of the monitoring phase. In the following sections, we will first explore how DL is applied to improve InSAR data processing and then focus on how it supports the various stages of monitoring and analysis.

DL models are applied at two distinct points in the workflow: first, to process and enhance the raw InSAR data (Processing), and second, to analyze the resulting data for monitoring tasks (Analysis).

**Figure 3:**
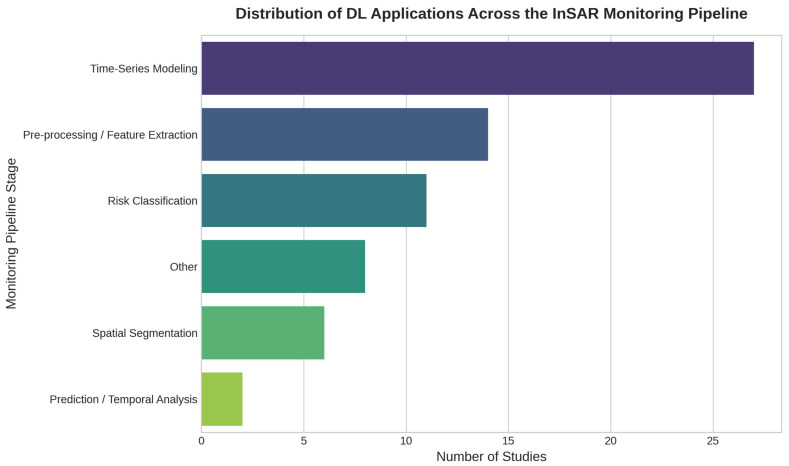
Distribution of DL applications across stages of the InSAR monitoring pipeline. The chart reveals a strong research focus on time-series modeling over other stages. It shows the number of studies applying DL models to different phases of the monitoring process, including: (i) Pre-processing (e.g., atmospheric correction, phase unwrapping); (ii) Time-series modeling (e.g., deformation trend analysis); (iii) Spatial segmentation and deformation detection; and (iv) Risk classification. Multi-stage applications are counted in all relevant categories.

A critical application of DL, though under-represented (accounting for only about 21% or 14 studies focusing on pre-processing/feature extraction according to Figure 3), is in the pre-processing stage. Here, models are used to correct inherent data limitations, such as mitigating atmospheric noise [64,81], which is a primary source of error in InSAR measurements. CNNs and DNNs are applied to reconstruct the atmospheric delay phase [64,81,89,91]. The quantitative impact of these corrections is significant: studies report that DNNs can reduce the standard deviation (STD) of deformation by 71% [89] or the STD of the phase by approximately 70% [91].

Other key processing tasks include algorithm-assisted phase unwrapping [30] and spatio-temporal interpolation [88]. In this latter task (interpolation), hybrid CNN-LSTM models have been used to create unified velocity maps, demonstrating a root mean square error (RMSE) reduction of over 73% [88]. CNNs are the prevalent architecture in this phase due to their strong image-based learning capabilities, although this application area remains less explored compared to analysis tasks.

### 4.3. DL Applications in Monitoring and Analysis (RQ2)

Building upon the advancements made in InSAR data processing (Section 4.2), the following subsections explore how DL is applied to monitor and analyze the processed data in specific infrastructure contexts, such as time-series modeling, spatial segmentation, and risk classification.

#### 4.3.1. Time Series Modeling and Deformation Prediction

This is the most frequently addressed stage in the literature, representing roughly 40% (27 studies) of the applications identified in Figure 3, focusing on the temporal evolution of deformation. Recurrent networks, primarily LSTM and GRU, are dominant for tasks such as univariate and multivariate deformation prediction [28,50,55], anomaly detection [37,92], and long-term forecasting, sometimes in hybrid configurations with statistical methods like ARIMA [50,55,92]. The effectiveness of these models is demonstrated in benchmarks against other approaches; for example, one study demonstrated that an LSTM reduced the RMSE by 51% in settlement prediction compared to a Random Forest (RF) model [93]. Attention-enhanced LSTMs are also used for temporal salience extraction [78], and emerging Transformer models show potential, with one study reporting a Mean Absolute Error (MAE) reduction of at least 58% [84].

#### 4.3.2. Spatial Segmentation and Deformation Detection

This stage focuses on identifying spatial zones with active deformation or instability, addressed in about 8% (5 studies) of the reviewed works (Figure 3). The tasks are dominated by computer vision architectures. CNNs and U-Net variants are widely used for pixel-level segmentation of deformation zones [42,45,63,74]. For object-based mapping and hazard detection, detectors such as YOLO [30,53,65] and Mask R-CNN [34,86] are employed. Performance in these studies is often reported in terms of computational efficiency: U-Net models report inference speeds of 80.4 FPS [45], Mask R-CNN achieves recognition speeds of 72.3 km^2^/s [86], and some lightweight YOLO models have as few as 2.26M parameters [65]. Segmentation models are increasingly equipped with attention modules to boost spatial accuracy under low coherence conditions [85].

#### 4.3.3. Risk and Susceptibility Classification

Classification of infrastructure vulnerability is addressed in approximately 16% (11 studies) of the corpus (Figure 3) using MLPs, DNNs, and ANNs [44,70,73], often in combination with non-DL classifiers (e.g., CatBoost, RF, SVM). These models generate susceptibility maps and help quantify structural exposure to geotechnical hazards [33,71].

#### 4.3.4. Multi-Stage and End-to-End Pipelines

Some studies [30,85] stand out by integrating multiple stages (e.g., pre-processing, segmentation, and prediction) into unified architectures, pointing to a trend toward operational end-to-end systems.

To synthesize how DL models are integrated into the InSAR-based monitoring process, Figure 3 summarizes the distribution of studies according to the phase of the pipeline in which the models are applied. The chart highlights a strong concentration in time-series modeling and deformation prediction tasks, whereas pre-processing and risk classification remain relatively underrepresented. These patterns suggest opportunities for expanding DL integration across the entire monitoring workflow.

### 4.4. Infrastructure Types Monitored (RQ3)

**Figure 4:**
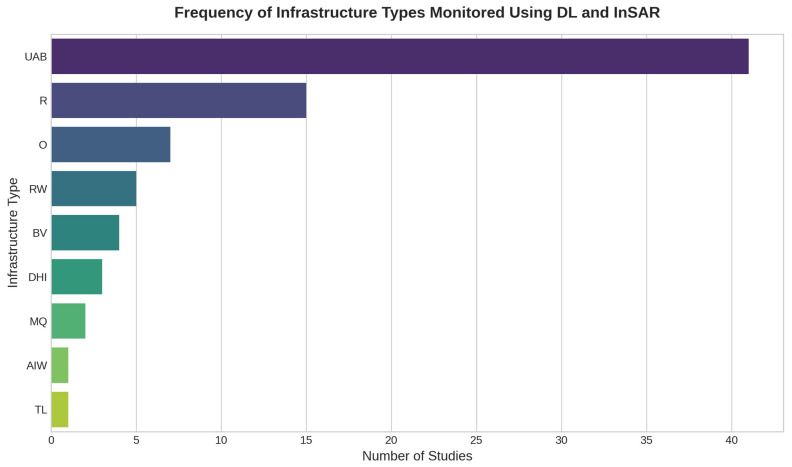
Frequency of infrastructure types monitored in the reviewed DL-InSAR literature. The chart aggregates the types of infrastructure across all 67 studies, counting each mention in multi-type studies separately. The results reveal a significant thematic imbalance, with a strong concentration of research on Urban Areas and Buildings (UAB) and Roads (R), while other critical infrastructures like dams and bridges remain substantially under-investigated.

The application domains of DL-InSAR reveal a significant thematic imbalance, as illustrated in Figure 4. Research has predominantly focused on Urban Areas and Buildings (UAB), which feature in 42 studies (approximately 63% of the corpus). This concentration is likely driven by factors such as the high density of potential Persistent Scatterers (PS) in built environments, the availability of extensive SAR datasets over cities, and the direct relevance to population risk management. Within this domain, studies commonly address challenges like subsidence monitoring [28,63], the detection of anomalous displacements affecting individual buildings or heritage structures [41], and assessing urban geotechnical risks.

Following urban settings, Linear Infrastructure constitutes the second most frequent application area. Roads (R) are monitored in 15 studies (22%) and Railways (RW) in 5 studies (7%). Their susceptibility to ground deformation (e.g., due to landslides or subsidence) necessitates continuous monitoring, making them suitable candidates for DL-InSAR approaches. Methodologies range from using CNNs for segmenting deformation patterns along road corridors [36], to employing LSTMs for predicting landslide-induced deformation affecting railways [61,83], and utilizing object detection models like Mask R-CNN or YOLO for hazard zone identification [34,65].

In contrast, other categories of critical infrastructure remain substantially under-investigated. Bridges and Viaducts (BV), despite being structurally sensitive assets often exposed to dynamic loads and settlement risks, appear in only 4 studies (6%) [54,55,77,94]. These works explore DL for anomaly detection and time-series modeling, sometimes integrating exogenous variables like temperature to enhance robustness [55]. Similarly, Dams and Hydropower Infrastructure (DHI), which pose significant potential hazards, are addressed in just 3 studies (4%) [45,89,90], primarily focusing on slope stability in surrounding areas or leveraging DL for atmospheric correction to improve data quality over reservoirs [89]. Other specific scenarios, such as Mines and Quarries (MQ) (3 studies, 4%) [52,57], Transmission Lines (TL) (1 study) [87], and Artificial Islands (AIW) (1 study) [84], are only marginally represented. Notably absent are studies focusing on tunnels, ports, or industrial facilities. Monitoring challenges in specific environments like permafrost regions or complex mountainous terrains are also highlighted, often requiring tailored model architectures [58,62].

## 5. Discussion

This review highlights the rapid growth and increasing sophistication of DL methods applied to InSAR data for infrastructure monitoring. It identifies not only consolidated methodological directions, but also critical limitations and opportunities that must be addressed to advance toward operational maturity.

To explore these findings, the discussion is organized by first assessing the methodological maturity along the main stages of the InSAR pipeline, from data processing to analytical modeling. Subsequently, we address the critical thematic and physical challenges that arise from the different application scales; namely, the distinction between large-scale territorial monitoring and focused structural monitoring. The section concludes by identifying emerging trends and future research opportunities that aim to bridge these gaps.

### 5.1. Methodology Maturity and Challenges in Data Processing

InSAR data processing involves several critical challenges, such as atmospheric noise, phase unwrapping, and computational complexity in handling large datasets. DL has emerged as a promising solution to these issues, but its applicability is still evolving. While significant strides have been made in reducing atmospheric noise and speeding up phase unwrapping, challenges persist in terms of data quality and the generalizability of these models across different environments.

To mitigate atmospheric noise, DL techniques have been successfully applied, particularly CNNs and GANs. For instance, CNNs have demonstrated a 71% reduction in the standard deviation (STD) of deformation signals by reducing atmospheric noise, leading to more accurate InSAR data [88]. Additionally, phase unwrapping, which has long been a computationally expensive task, has been accelerated with CNN-based approaches, reducing processing time and increasing inference speeds to as high as 80.4 FPS [45]. These solutions represent significant progress in overcoming data processing bottlenecks. However, while these techniques have yielded impressive results, they still face limitations related to data generalization. Most DL models are trained on case-specific datasets, and a lack of standardized, open-access datasets makes it difficult to compare and evaluate models across different regions and infrastructures.

As identified in Section 4.2, the first application of DL in the pipeline is Data Processing. This phase, which includes atmospheric noise correction [64,81], phase unwrapping [30], and interpolation [88], is critical as it directly impacts the quality of all subsequent analyses. In [30,64], for example, CNNs are applied to pre-process raw InSAR signals. The quantitative results reported in Section 4.2 are promising, demonstrating that DL models can effectively tackle these complex, non-linear tasks (e.g., STD reductions of ~70% [89,91] or RMSE reduction over 73% [88]).

However, this methodological area remains critically under-explored (Figure 3) and suffers from a foundational limitation: the Lack of Standardized Training Data. Many models are trained on case-specific or synthetic data [33,45,73], preventing broad generalization. No study proposes an open, labeled benchmark dataset for tasks like atmospheric correction. This lack of standardization makes robust model comparison difficult. For instance, it is unclear whether the 71% STD reduction reported in [89] is directly comparable to the ~70% reduction in [91], as these results were achieved on different datasets and validation contexts. Without a common validation dataset, assessing true model transferability or performance remains a significant challenge.

This challenge is intrinsically linked to Data Quality and Noise Handling. While CNN-based denoising is a common theme [30,64,86], the application lacks standardization. This validation gap forces a shift in how performance is evaluated. Without common accuracy benchmarks, studies often pivot to evaluating computational efficiency as a proxy for operational readiness. Performance is thus reported in metrics like inference speed (e.g., 80.4 FPS for a U-Net model [45]) or area processed per second (e.g., 72.3 km^2^/s for a Mask R-CNN [86]), while others simply note a “significant time reduction” [33] as the primary benefit.

While operational speed is important, this focus on efficiency sidesteps the core challenge of validating the correctness of the processing. If the denoising or phase unwrapping model (e.g., [30]) introduces subtle artifacts, any subsequent analysis model (like an LSTM for prediction) will be trained on flawed data. This lack of validated, reproducible processing pipelines undermines the foundation upon which all monitoring and analysis tasks are built, highlighting a critical gap in the literature.

While the improvements in data processing set the foundation for accurate monitoring, challenges still exist in ensuring that the data is suitable for subsequent analysis and interpretation, particularly in the context of complex infrastructural monitoring. The lack of standardized datasets remains a foundational limitation. Many models are trained on case-specific or synthetic data, preventing broad generalization. No study proposes an open, labeled benchmark dataset for tasks like atmospheric correction or phase unwrapping, making it difficult to compare models directly. This lack of standardization makes robust model comparison difficult, which undermines the foundation upon which all monitoring and analysis tasks are built.

### 5.2. Methodology Maturity and Challenges in Monitoring and Analysis

As shown in Figure 3, the vast majority of reviewed studies apply DL to Monitoring and Analysis tasks (Section 4.3). This is where methodological maturity appears highest and the diversity of architectures is greatest. A prominent trend is the predominance of LSTM networks, applied in more than 20 studies [28,29,31,32,41,47,50,51,55,58,59,61,67,77,78,79,80,82,83,88,92,93] for forecasting deformation time series. Their popularity is justified by strong quantitative performance against other methods, such as an LSTM reducing RMSE by 51% compared to a Random Forest (RF) model in settlement prediction [93].

**Figure 5:**
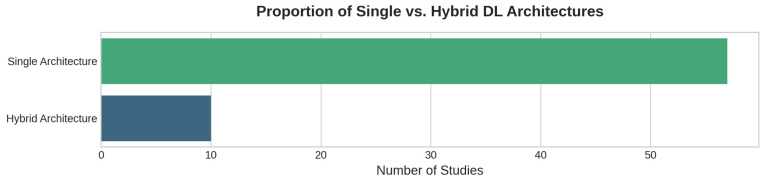
Proportion of hybrid versus single DL architectures used in InSAR-based infrastructure monitoring. Hybrid models are present in only a small subset of studies, reflecting a methodological fragmentation that constrains the evolution of end-to-end monitoring systems.

Alongside time-series modeling, CNNs feature prominently [30,31,32,34,36,38,39,40,43,47,48,49,52,60,66,68,70,72,74,75,85,86], primarily for the analysis tasks of spatial segmentation (Section 4.3.2) and feature extraction. U-Net architectures, while not always explicitly named, are a common choice for this [30,85]. More sophisticated implementations combine these components into hybrid (e.g., CNN-LSTM) architectures [31,32,39,40,47,48,80] to capture complex spatiotemporal dependencies, though these remain in the minority (Figure 5).

Another important trend is the emergence of attention-based models, including Transformer architectures [38,78,84,85,90,94]. These aim to improve model focus on relevant spatiotemporal patterns and show promise, with one study reporting a Mean Absolute Error (MAE) reduction of at least 58% [84].

**Figure 6:**
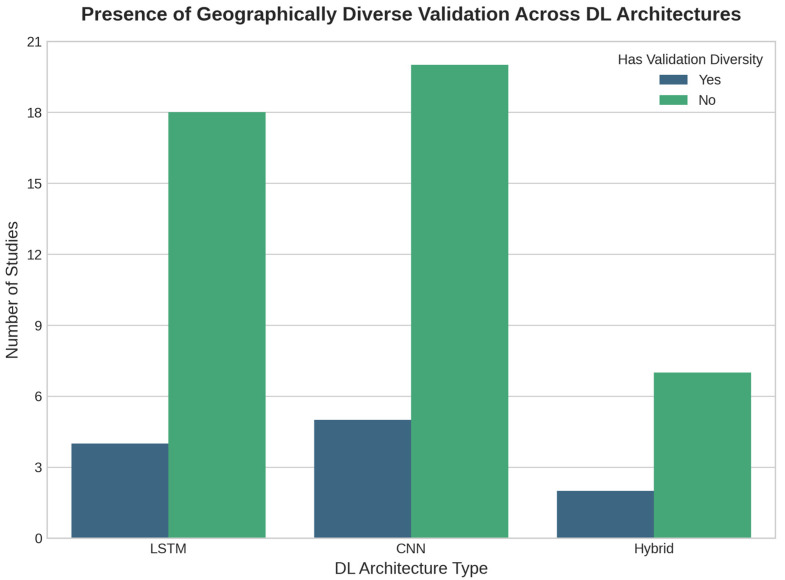
Presence of geographically diverse validation across DL architectures. The figure shows the number of studies that apply each architecture type (LSTM, CNN, Hybrid) and whether validation is performed in multiple infrastructure or geographic contexts. The absence of diverse validation scenarios suggests a gap in assessing model generalizability and robustness.

Despite this progress, these analysis models suffer from critical limitations. Many studies show Insufficient Performance Evaluation, often relying on standard metrics (e.g., accuracy, RMSE) without considering engineering-relevant thresholds or robust cross-validation. This leads to the most significant challenge: Poor Generalization (as shown in Figure 6). The overwhelming majority of models are validated only in a single geographic area or infrastructure type, which is problematic for real-world applications.

Furthermore, most models lack explainability mechanisms (XAI), such as saliency maps or attention visualizations [62,78,85]. More advanced XAI techniques (e.g., SHAP, LIME, Grad-CAM), specifically adapted to InSAR signals, are absent. This lack of interpretability hampers trust and practical adoption. This review argues that the “Poor Generalization” problem is not merely a data-scaling issue but a fundamental conceptual flaw. It arises from the literature’s tendency to apply models trained for one context (e.g., slow, large-scale subsidence) to another (e.g., rapid, localized structural deformation) without considering the different underlying physics. This critical distinction is explored in the following section.

The successful application of DL in monitoring and analysis will rely heavily on advancements made in data processing. Therefore, overcoming challenges in data quality and model interpretability will be essential to fully realize the potential of DL in infrastructure monitoring.

### 5.3. Physical Limitations and the Territorial-Structural Distinction

The “Poor Generalization” challenge discussed in the previous section is directly linked to the thematic imbalance observed in the results (Section 4.4, Figure 4). The literature is heavily concentrated on Territorial-Scale Monitoring, such as urban subsidence [28,63] and landslides [36,61,83]. Urban Areas and Buildings (UAB) alone appear in 42 of the 67 reviewed studies, followed by Roads (R) and Railways (RW). In these scenarios, deformation is typically slow (mm/year), cumulative, and spatially extensive. The main DL challenge is modeling complex, non-linear temporal dependencies over long periods. Recurrent architectures like LSTMs are well-suited for this task, as they are designed to capture long-term patterns.

Conversely, Structural-Scale Monitoring, which focuses on specific assets like Bridges and Viaducts (BV) [54,55,77,94] or Dams and Hydropower Infrastructure (DHI) [45,89,90], is significantly underrepresented. This review argues that this is not merely a “thematic gap” but a barrier rooted in the physical limitations of InSAR. The challenges in structural monitoring are fundamentally different: engineered structures, particularly steel bridges, are subject to rapid, cyclical, and high-magnitude deformations, often driven by thermal expansion.

This distinction exposes a critical physical limitation of InSAR that most DL studies ignore. The maximum detectable displacement between two radar acquisitions is constrained by the phase ambiguity limit, typically λ/4 of the sensor’s wavelength. As demonstrated in a recent study [95], this limit is approximately 1.4 cm for C-band satellites (e.g., Sentinel-1) and only 0.8 cm for X-band (e.g., COSMO-SkyMed).

In a study of a steel bridge [95], it was found that the daily thermal expansion of the deck, driven by temperature changes, frequently exceeded this λ/4 limit. This resulted in the satellite data (particularly X-band) showing low coherence and “a substantial inability” to measure the true displacement, a phenomenon known as aliasing. The satellite signal was decorrelated before the deformation could even be properly measured.

This implies that many DL models applied to structural monitoring (e.g., [54,55]) may be fundamentally flawed; they are potentially being trained on aliased signals or decorrelation noise, not the true, high-frequency physical deformation. DL methods trained exclusively on territorial data (e.g., urban subsidence) cannot be naively transferred to geotechnically complex structural settings. This highlights that scenario-specific signal characteristics (e.g., thermal cycles in bridges, hydrological cycles in dams) are not just minor variables but core physical constraints that must be addressed, for example, through the Integration of Auxiliary and Contextual Data (such as thermal or hydrological information) [55,62,90].

### 5.4. Emerging Trends and Future Research Opportunities

Despite the methodological and physical limitations identified, the literature reveals several promising developments that signal a maturing field. These advances are particularly relevant in addressing spatiotemporal complexity, solving the physical-signal challenges (Section 5.3), enhancing model performance, and moving toward integrated and trustworthy systems.

Architecturally, the field is moving beyond simple models. Several studies implement Hybrid and Multi-Branch Architectures to handle distinct aspects of the InSAR signal. For example, refs. [31,32,39,40,47,80] employ CNN-LSTM or CNN-BiGRU configurations, where convolutional layers extract spatial features and recurrent layers capture temporal evolution. These models outperform simpler architectures in complex tasks such as landslide detection [40]. Notably, refs. [48,80] extend this approach by integrating multi-branch networks for simultaneous analysis of different input sources or spatial resolutions, aligning with the multiscale nature of InSAR data. Alongside hybrids, Attention Mechanisms and Transformers are a growing trend [38,78,84,85,90,94]. Attention blocks (e.g., CBAM) [42,62] highlight salient features, while self-attention [78,85] can detect key temporal events. Transformer-based models [84,90] offer scalability for long, irregular sequences, suggesting suitability for dynamic structural scenarios such as bridges [84].

Perhaps more critically, as a direct response to the physical limitations identified in Section 5.3, is the Integration of Auxiliary and Contextual Data. Several studies are exploring data fusion, integrating InSAR time series with auxiliary variables such as meteorological data (e.g., precipitation, temperature) [31,38,84], geotechnical or hydrological information [55,62,90], or topographic context [48,84]. This is not merely a trend, but a requirement for robust structural monitoring. For example, ref. [55] combines thermal and hydrological signals to forecast deformation in dams, helping to capture the causal relationships behind observed deformations. This use of context-aware models bridges the gap between purely data-driven methods and expert-informed geotechnical understanding. This naturally leads to the need for physics-informed DL models, which would integrate geophysical or geotechnical principles (e.g., hydromechanical behavior or thermal constraints [95]) directly into the learning process.

To move toward operational maturity, the field must also address the validation and trust deficits identified in Section 5.1 and Section 5.2. An immediate priority is the creation of standardized, open-access benchmark datasets. As noted, the lack of reproducibility and proprietary data makes it difficult to compare models fairly. Open datasets covering diverse geographies and infrastructure types are needed to accelerate methodological innovation. Closely tied to this is the need for standardized evaluation protocols that move beyond simple metrics (e.g., RMSE) and consider engineering-relevant thresholds and uncertainty quantification. Furthermore, a major challenge is explainability (XAI). As discussed, DL models remain largely black-box systems. Incorporating XAI (e.g., SHAP, LIME, Grad-CAM) is essential for building trust among infrastructure managers, particularly in safety-critical contexts.

Finally, the long-term vision involves integrating these robust, explainable models into scalable, real-time systems. Some studies (e.g., [30,85]) propose modular, end-to-end pipelines that reduce human intervention and move toward deployment in early warning systems. This requires research into computational efficiency under large-scale data streams and the implementation of operational alert thresholds. This vision culminates in fusion with digital twin frameworks, enabling predictive simulation, real-time feedback, and proactive maintenance planning. This requires advances not only in model accuracy but also in interoperability with GIS, BIM, and other engineering data environments.

**Figure 7:**
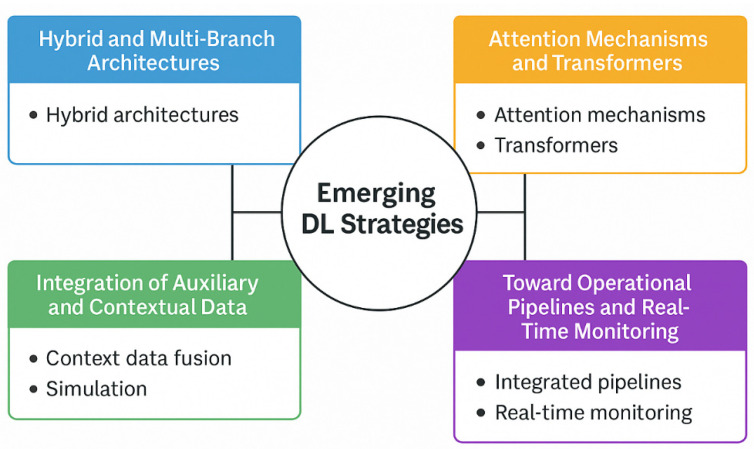
Emerging DL strategies in the literature on InSAR-based infrastructure monitoring. The diagram organizes innovation paths into four axes: (i) architectural design (hybrid models, attention, transformers), (ii) contextual data integration (geotechnical, environmental, simulation), (iii) operational implementation (real-time pipelines, scalability), and (iv) interpretability (XAI, saliency maps, trust mechanisms). These areas represent the most relevant opportunities for advancing DL-InSAR systems toward real-world applicability.

**Figure 8:**
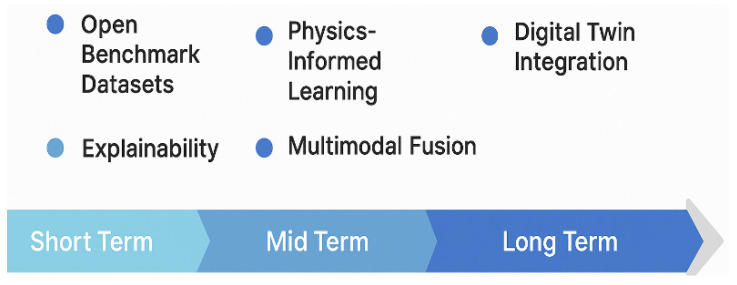
Research roadmap for DL-InSAR infrastructure monitoring. The timeline presents short-, mid-, and long-term research priorities for advancing DL applications in InSAR-based infrastructure monitoring. Short-term goals emphasize the creation of open benchmark datasets and the adoption of explainability mechanisms. Mid-term priorities include the integration of physics-informed learning and multimodal data fusion. Long-term objectives focus on scalable deployment and full integration with digital twin platforms for real-time decision support.

These directions have the potential to bridge the gap between academic innovation and real-world application, unlocking the full potential of DL-InSAR methodologies. To visually consolidate these innovation directions, Figure 7 presents a strategic map of emerging DL approaches. Figure 8 structures these challenges and opportunities along a temporal and technological horizon, summarizing the research roadmap for the field.

### 5.5. Synthesis Across Research Questions

The research questions (RQs) defined at the outset of this review provided a framework for systematically assessing the current state of the art in DL-based infrastructure monitoring using InSAR data. The findings discussed in Section 4 and Section 5 allow for the synthesis presented in Table 2.

## 6. Conclusions

The integration of DL with InSAR data represents a promising frontier for the monitoring of critical infrastructure. This systematic review analyzed 67 studies published between 2020 and 2025, identifying current trends, methodological practices, thematic priorities, and outstanding challenges in the field.

The analysis shows that DL has been primarily applied in time-series deformation modeling and spatial segmentation. LSTM and CNN architectures dominate the literature, with a growing number of studies exploring hybrid and attention-based models. However, methodological fragmentation remains evident. Many models are task-specific, trained on case-sensitive datasets, and lack transparency, benchmark validation, or interpretability mechanisms.

From an application perspective, the literature is heavily concentrated on urban settings and linear transport infrastructure, especially roads and railways. Other critical domains, such as dams, tunnels, bridges, and offshore platforms, are substantially underexplored. This thematic imbalance limits the generalizability of existing approaches and restricts the scope of DL-InSAR applications to the most data-rich environments.

Despite these limitations, the field is evolving. Recent studies have introduced modular pipelines, fused InSAR with environmental and geotechnical variables, and experimented with novel architectures such as Transformers and physics-informed DL. These innovations reflect a growing maturity and suggest a pathway toward real-time, scalable, and interpretable infrastructure monitoring systems.

Based on these findings, the review identifies six strategic research priorities:

1. the development of open and standardized benchmark datasets,
2. the adoption of explainable AI frameworks tailored to InSAR,
3. the integration of contextual and multimodal data,
4. the design of end-to-end architectures with operational capacity,
5. the incorporation of physics-informed constraints, and
6. the alignment of DL models with digital twin environments and early warning systems.

By consolidating the current state of research and outlining a future research agenda, this review provides a structured foundation for researchers, engineers, and policymakers aiming to advance the use of DL-InSAR systems in infrastructure risk monitoring. Addressing the identified gaps will be essential to ensure that these models are not only accurate, but also robust, transparent, and operationally useful.

Future research should focus on improving the interpretability of DL models in InSAR applications. This will be crucial for operational adoption in high-risk infrastructure sectors, where decisions are often made based on model outputs. Furthermore, the integration of DL-InSAR systems into real-time monitoring frameworks and digital twin technologies will revolutionize preventative maintenance, allowing for proactive measures in the management of critical infrastructure.

## Figures and Tables

**Table 1:** Summary of the key characteristics of the 67 studies included in the review. For each study, the table reports the DL architecture used, the type of infrastructure monitored, the InSAR variant applied, the monitored phenomenon, and a summary of its key contribution or finding. This synthesis allows for the identification of methodological trends, such as the prevalence of LSTM and CNN architectures, and thematic imbalances, like the focus on urban areas (UAB) and landslides (LS). The abbreviations used throughout the table are explained below.

Ref.	DL Architecture	Monitored Infrastructure	InSAR Type	Monitored Phenomenon	Input	Output	Computational Complexity
[28]	LSTM	UAB	GB-InSAR	LS	TS (Def + Ext)	Pred: Future Def. and Atmos. Delay	Time (inf): 4.73 min/scene
[29]	LSTM; TGLSTM	UAB	PS-InSAR	LS, SF, IM, SB	TS (Def)	Detect: Change Point Prob.	Time (inf): 15 min/630 k series
[30]	U-Net; YOLOv3; DnCNN	O, UAB	InSAR	LS, DM	Img (Phase + Coh + Amp)	Seg/Detect/Recon: Multistage Pipeline	Qual: ‘Detection-First’ for efficiency
[31]	CNN; LSTM	O	InSAR	SB	Img/TS (Def + Ext)	Pred: Future Def. Image (6-day)	Qual: Computationally efficient
[32]	CNN; LSTM; GRU; SRU	UAB	SBAS-InSAR	LS	FV (Factors + Def)	Class: Suscept. Map	Qual: SRU for training speed
[33]	DMLP; LSM	UAB	MT-InSAR	LS	FV (Factors + Def)	Class: Suscept. Map	Qual: Significant time reduction
[34]	Mask R-CNN	R	InSAR	LS	Img (Vel)	Seg: Instance Mask	Params: 32.18 M; FLOPS: 298.53 M
[35]	SOM	UAB	MT-InSAR	IM	TS (Def)	Detect: Alert Signal (Cluster-based)	Qual: Real-time big data processing
[36]	CNN	R	InSAR	LS	Img (Phase Rate + Def + DEM + Slope)	Seg: Binary Mask (Active Areas)	Qual: Robust and efficient method
[37]	Autoencoder	UAB	SBAS-InSAR	LS	TS (Def + Ext)	Pred: Future Def.	Time (train): 772 s/epoch
[38]	CNN; YOLOv5; Transformer	R, UAB	InSAR + DEM (SBAS)	LS	Img (Composite Feature)	Detect: Bounding Box	Qual: Efficient input feature (GRCI)
[39]	CNN; Bi-GRU	R, O	SBAS-InSAR	LS	TS (Def + Ext)	Class: Suscept. Probability	Time (train): ~1 h 40 min
[40]	CNN; Bi-GRU	UAB	MT-InSAR/SBAS	LS	TS (Def + Ext)	Pred: Future Def.	Time (inf): 8–10 min/scatterer
[41]	Autoencoder; LSTM	UAB	PS-InSAR	IM	TS (Def)	Class: Anomaly Type	Qual: RRCF benchmark reduces costs
[42]	U-Net	R, UAB	InSAR	LS	Img (Phase Gradient)	Seg: Binary Mask	Qual: Computationally efficient
[43]	CNN	R	InSAR	LS	FV (Factors + Def Trend)	Class: Potential Landslide (Binary)	Qual: Efficient, saves interpretation time
[44]	DNN	R, UAB	SBAS-InSAR	LS	FV (Factors + Def + Coords)	Class: Active Landslide (Binary)	NR
[45]	U-Net	DHI	SBAS-InSAR	LS	Img (Vel)	Seg: Deformation Zone Mask	FLOPS: 11.48 G; Speed (inf): 80.4 FPS
[46]	U-Net	UAB	MT-InSAR	DM	Img (Coh + Infra. Map)	Pred: PS Count	Time (inf): 2 s/1320 km^2^
[47]	CNN; LSTM	UAB	IPTA	DM	TS (Def)	Pred: Future Def.	Qual: Pre-processing reduces load
[48]	CNN; RNN	UAB	SBAS-InSAR/PS-InSAR	LS	FV (Factors)	Class: Suscept. Map	Train Params: BS 8/64, Epochs 250/500
[49]	CNN	UAB	SBAS-InSAR	LS	Img (Factors + Def + Optical)	Seg: Recognition Mask	NR
[50]	LSTM; ARIMA	O	InSAR	DM	TS (Def)	Pred: Future Def.	Train Params: 100 Epochs, BS 128
[51]	LSTM	R, UAB	SBAS-InSAR	LS	TS (Def + Ext)	Pred: Future Def.	NR
[52]	CNN	RW, MQ, O, UAB	PS-InSAR	IM	Img (Vel)	Class: Prob. Map	Qual: Scalable framework
[53]	YOLOv3	UAB	InSAR	LS	Img (Phase Gradient)	Detect: Bounding Box	Qual: YOLOv3 for faster inference
[54]	ANN	BV	InSAR	BM	TS (Def)	Detect: Anomaly Score	Qual: MSD chosen for simplicity
[55]	LSTM; SARIMA; Prophet	BV	PS-InSAR	BM	TS (Def + Ext)	Pred: Future Def.	Time (train): Seconds to minutes
[56]	GCN; GRU	UAB	SBAS-InSAR	LS	Graph (Nodes as Def points)	Pred: Future Def. (per node)	Qual: STGCN requires more resources
[57]	DNN	MQ	InSAR	IM	FV (Meta)	Pred: Future Def.	Qual: Simple arch. outperformed complex ones
[58]	LSTM	RW	MT-InSAR/SBAS	DM	TS (Def)	Pred: Future Def.	Qual: LSTM hard to parallelize
[59]	LSTM	O	InSAR	DM	FV (Creep Params)/TS (Def)	Pred: TS (train)/FV (final)	Qual: Overcomes slow lab tests
[60]	CNN; DNN	RW	InSAR Stacking	LS	FV (Factors)	Class: Suscept. Index	Time (train): DNN ~45 epochs; CNN ~1300 epochs
[61]	LSTM; RNN	R	SBAS-InSAR	LS	TS (Def)	Pred: Future Def.	Qual: Outperforms manual measurement
[62]	Bi-GRU	RW	SBAS-InSAR	DM	TS (Def + Ext)	Pred: Future Def. and Ext	Time (train): 40–55 epochs for convergence
[63]	U-Net	UAB	SBAS-InSAR	SB	SeqImg (Def)	Pred: Future Def. Image	Params: 4.025 M
[64]	CNN	UAB	InSAR	LS	FV (DEM + Coords)	Recon: Atmos. Delay Phase	NR
[65]	YOLO (v3/v8)	UAB	SBAS-InSAR	LS	Img (Vel)	Detect: Bounding Box	Params: 2.26 M
[66]	CNN	UAB	InSAR	IM	Img (Coh Matrix)	Class: Land Cover Map	Qual: SVM (comparative) is efficient
[67]	LSTM; TCN	UAB	InSAR	LS	TS (Def)	Pred: Future Def.	Qual: TCN for efficient processing
[68]	CNN	UAB	SBAS-InSAR	LS	Img (Factors + Def)	Class: Landslide Probability	Qual: MCE-CNN reduces complexity
[69]	BP-ANN	UAB	SBAS-InSAR	LS	FV (Factors + Def)	Class: Suscept. Index	Qual: SSA-BP converges faster
[70]	CNN; RF; SVM; DBN	UAB	SBAS-InSAR	LS	FV (Factors)	Class: Landslide Probability	NR
[71]	MLP	UAB	MT-InSAR	LS	FV (Factors + Def)	Class: Landslide Probability	Qual: Few gradient updates reduce cost
[72]	CNN	UAB	InSAR	LS	FV (Factors + Def)	Class: Suscept. Index	Qual: Bayesian Opt. for speed
[73]	ANN; CatBoost	R	SBAS-InSAR	LS	FV (Factors + Def)	Class: Hazard Suscept. Prob.	NR
[74]	CNN	UAB	InSAR	LS	Img (Phase + Sin(Phase) + Cos(Phase))	Seg: Semantic Mask	Params: 72.24 M; FLOPS: 67.91 G
[75]	CNN	UAB	InSAR + TCP	LS	TS (Def + Ext)	Interp: High-freq. daily Def.	Qual: Upsamples from 12-day to daily
[76]	ANN	UAB	MT-InSAR, MG	IM	TS (Def)	Detect/Class: Velocity Anomaly	Speed (inf): 2.5 M samples/s; Time (inf): 68 s/170 M series
[77]	LSTM	R, BV	InSAR + GRACE-FO	SB	TS (Def)	Pred: Future Def. (6-day)	NR
[78]	LSTM + Attention	UAB	SBAS-InSAR	IM	TS (Def)	Pred: Future Def.	NR
[79]	RNN; GRU; LSTM	UAB	SBAS-InSAR	LS	FV (Factors + Def)	Class: Suscept. Map	NR
[80]	ConvLSTM	R, UAB	Dual-pol MT-InSAR	SB	SeqImg (Def)	Pred: Future Def. Image	Qual: Significantly improves efficiency
[81]	CNN	UAB	InSAR	AN	Img (Phase + DEM)	Recon: Atmos. Noise Map	Qual: Correction at native resolution
[82]	LSTM	R	SBAS-InSAR	SB	TS (Def)	Pred: Future Def.	NR
[83]	LSTM	RW	SBAS-InSAR	RWM	TS (Def)	Pred: Future Def.	NR
[84]	Transformer	AIW, BV	TS-InSAR	BM	TS (Def, synthetic)	Decomp: Trend and Seasonal Comp.	Qual: Reduced MAE by at least 58%
[85]	CNN; Transformer	UAB	SBAS-InSAR	LS	Img (Def + Coh + DEM)	Class: Hazard Level (Multi-class)	NR
[86]	Mask R-CNN	UAB	InSAR	LS	Img (Vel)	Seg: Instance Mask	Speed (inf): 72.3 km^2^/s (recognition)
[87]	MLP	TL	SBAS-InSAR	SB	FV (Factors)	Class: Suscept. Map	NR
[88]	LSTM; CNN	R, UAB	MT-InSAR	LS	TS / Img (Def)	Interp: Unified Velocity Map	Qual: RMSE decreased by >73%
[89]	DNN	DHI	InSAR	LS	FV (DEM + Coords + Coh)	Recon: Atmos. Delay Phase	Qual: STD of deformation reduced 71%
[90]	Transformer	DHI	SBAS-InSAR	LS	TS (Def + Ext)	Pred: Future Def. (Long-term)	Qual: Complexity O(L log L)
[91]	DNN	UAB	TS-InSAR, SBAS-InSAR	LS	FV (Phase + DEM + Coords + Coh)	Recon: Atmos. Delay Phase	Qual: STD of phase reduced ~70%
[92]	LSTM; Seq2Seq; SARIMA	UAB	InSAR	DM	TS (Def, uni- or multivariate)	Pred: Future Def. (1–9 months)	Qual: Performance comparison (RMSE)
[93]	LSTM	O	InSAR, SBAS-InSAR, PS-InSAR	SB	TS (Def)	Pred: Future Settlement	Qual: Reduced RMSE by 51% vs. RF
[94]	Self-Attention Model	R, UAB	TS-InSAR, SBAS-InSAR, PS-InSAR	LS	Img (Vel, with prompts)	Seg: Instance Mask (Zero-shot)	Qual: Method described as “extremely fast”

Monitored infrastructure: UAB = Urban Areas and Buildings; R = Roads; DHI = Dams and Hydropower Infrastructures; O = Others; RW = Railways; MQ = Mines and Quarries; BV = Bridges and Viaducts; TL = Transmission Lines; AIW = Artificial Islands and Walls. Monitored phenomenon: LS = Landslide; DM = Deformation Monitoring; SB = Subsidence; IM = Infrastructure Monitoring; SF = Seismic Faults; ANP = Atmospheric Noise Pre-processing; BM = Bridge Monitoring; AN = Atmospheric Noise; RWM = Roadway and Railway Monitoring. Input Data Form: TS = Time Series; Img = Image/Patch; FV = Feature Vector; Graph = Graph Data; SeqImg = Sequence of Images. Input Data Content: Def = Deformation; Vel = Velocity; Phase = Phase/Interferogram; Coh = Coherence; Amp = Amplitude; Ext = External Factors (e.g., climate, hydrology); Factors = Susceptibility/Thematic Factors; DEM = Digital Elevation Model; Coords = Coordinates; Meta = Metadata. Output Task and Result: Pred = Prediction (e.g., Future Def., PS Count); Class = Classification (e.g., Suscept. Map, Hazard Level); Seg = Segmentation (e.g., Instance Mask, Binary Mask); Detect = Detection (e.g., Bounding Box, Anomaly Score); Recon = Reconstruction (e.g., Atmos. Delay Phase); Decomp = Decomposition (e.g., Trend and Seasonal Comp.); Interp = Interpolation (e.g., High-freq. daily Def.). Computational Complexity: Time (train) = Training Time; Time (inf) = Inference Time; Params = Model Parameters; FLOPS = Floating Point Operations per Second; Speed (inf) = Inference Speed (e.g., FPS, samples/s); Train Params = Training Parameters (e.g., Epochs, Batch Size—BS); Qual = Qualitative Assessment; NR = Not Reported.

**Table 2:** Synthesis linking research questions to key insights. The table summarizes how each research question (RQ) is addressed across the reviewed literature, including main findings and representative examples. It consolidates the thematic and methodological observations discussed throughout the review.

Research Question	Key Findings	Illustrative References
**RQ1:** What DL models have been used in infrastructure monitoring with InSAR data?	LSTM and CNN are the dominant architectures, used for time-series modeling and spatial segmentation, respectively. Hybrid models (e.g., CNN-LSTM, CNN-BiGRU) are emerging but underused. Transformer and attention-based models show potential but are limited in number.	[28,31,39,40,78,84,85]
**RQ2:** At what stages of the monitoring process are InSAR data integrated with DL?	DL applications are split between data processing (e.g., denoising, atmospheric correction) and analysis (e.g., prediction, segmentation). Analysis tasks, especially time-series modeling, are far more common, while critical processing steps remain under-explored and lack standardized validation.	[30,42,50,64,85]
**RQ3:** What types of infrastructure have been most frequently monitored?	A strong thematic imbalance exists, favoring territorial-scale applications (urban areas, landslides) over structural-scale ones (bridges, dams). This imbalance is intrinsically linked to physical InSAR limitations (e.g., thermal decorrelation exceeding λ/4 limit in structural monitoring [95]), which most current DL analysis approaches fail to address, hindering generalization.	[28,36,52,54,55,90]

## Data Availability

No new datasets were generated. All data analyzed derive from published articles cited in the manuscript. The data-extraction approach is fully described in the Methods (Section 3.5). The extraction template and the consolidated table of extracted study characteristics can be provided by the corresponding author upon reasonable request.
